# Supplementary material for: The Construction and Exploration of a Comprehensive MicroRNA Centered Regulatory Network in Foxtail Millet (Setaria italica L.)
Source: Front Plant Sci. 2022 May 6;13:848474. doi: 10.3389/fpls.2022.848474 (PMC9121102; doi:10.3389/fpls.2022.848474)
Supplement: Supplementary file 7 [file Data_Sheet_7.zip › Sit-miRN3000_Seita.7G221200.1_1127_TPlot.pdf]

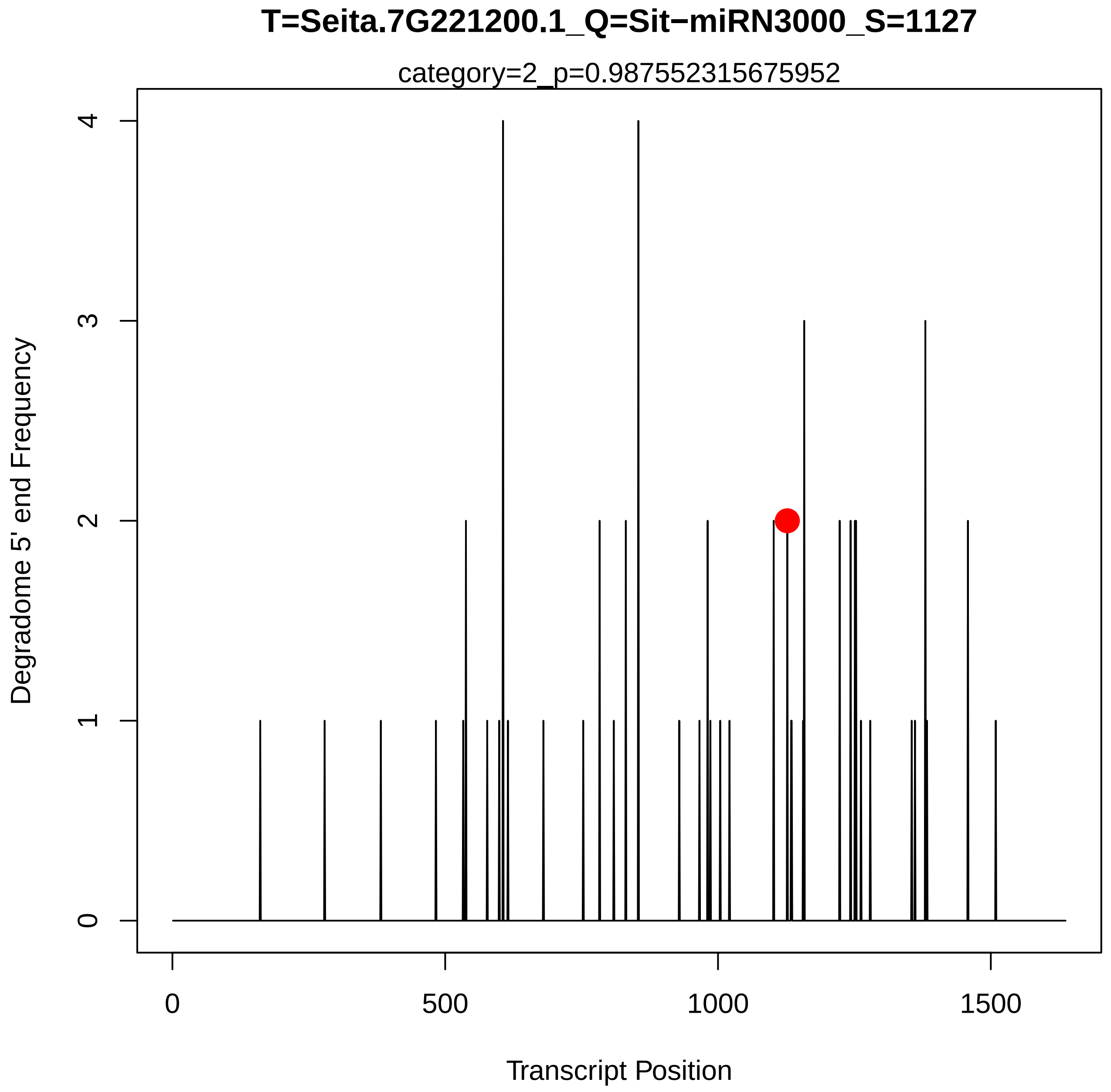
T=Seita.7G221200.1_Q=Sit−miRN3000_S=1127
category=2_p=0.987552315675952
Degradome 5' end Frequency
0
1
2
3
4
0
500
1000
1500
Transcript Position
